# Can Cognitive Control and Attentional Biases Explain More of the Variance in Depressive Symptoms Than Behavioral Processes? A Path Analysis Approach

**DOI:** 10.3389/fpsyg.2022.809387

**Published:** 2022-03-23

**Authors:** Audrey Krings, Jessica Simon, Arnaud Carré, Sylvie Blairy

**Affiliations:** ^1^Psychology and Neuroscience of Cognition Research Unit (PsyNCog), Université de Liège, Liège, Belgium; ^2^LIP/PC2S, Université Savoie Mont Blanc, Université de Grenoble Alpes, Chambéry, France

**Keywords:** behavioral activation, cognitive control training, depression, brooding, cognitive control

## Abstract

**Background:**

This study explored the proportion of variance in depressive symptoms explained by processes targeted by BA (activation, behavioral avoidance, anticipatory pleasure, and brooding), and processes targeted by cognitive control training (cognitive control, attentional biases, and brooding).

**Methods:**

Five hundred and twenty adults were recruited. They completed a spatial cueing task as a measure of attentional biases and a cognitive task as a measure of cognitive control and completed self-report measures of activation, behavioral avoidance, anticipatory pleasure, brooding, and depressive symptoms. With path analysis models, we explored the relationships between these predictors and depressive symptoms.

**Results:**

BA processes were significant predictors of depressive symptoms, and activation partially predicted anticipatory pleasure, which in turn predicted depressive symptoms. However, cognitive control and attentional biases predicted neither brooding nor depressive symptoms. A comprehensive model including all processes fit the data but did not explain more of the variance in brooding or depressive symptoms than a model including only BA processes.

**Limitations:**

The spatial cueing task was associated with low reliability and the use of a non-clinical sample limited the generalizability of the conclusions.

**Conclusion:**

Activation, behavioral avoidance, brooding, and anticipatory pleasure are relevant processes to target in order to reduce depressive symptoms, while cognitive control and attentional biases are not.

## Introduction

Depression is a very prevalent condition (World Health Organization, 2020) and one of the leading causes of disability worldwide (Kessler and Bromet, 2013). Although there are currently several evidence-based interventions for depression associated with a significant reduction of depressive mood, the rates of relapse and recurrence of depression remain high (Vittengl et al., 2007; Bockting et al., 2015). One possible explanation is that existing treatments do not sufficiently target vulnerability processes involved in the etiology and maintenance of depression. A combination of several existing treatments could be a promising way to improve depression care.

Inspired by behavioral models in psychology, behavioral activation (BA) aims to increase activation and reduce avoidance patterns in order to increase reinforcing experiences and consequently reduce depressive symptoms (Lejuez et al., 2001; Martell et al., 2001; Manos et al., 2010). Empirical data revealed that avoidance positively predicts depressive symptoms while activation negatively predicts them (Wagener et al., 2016). Moreover, brooding, perceived as frequent covert avoidance, positively predicts depressive symptoms (Nolen-Hoeksema et al., 2008; Watkins and Roberts, 2020). BA is a well-established empirical treatment that improves depressive symptomatology (Ekers et al., 2014; Cuijpers et al., 2020), wellbeing (Mazzucchelli et al., 2010), and quality of life (Orgeta et al., 2017) of clinically and subclinically depressed individuals. More specifically, empirical data show that BA improves activation (Dimidjian et al., 2017) and decreases avoidance (Krings et al., 2020) and brooding (McIndoo et al., 2016). In addition, preliminary research using fMRI found that depressed participants treated with BA showed decreased activation in the prefrontal neuronal structures involved in cognitive control (CC; Dichter et al., 2010), as well as improved functioning of appetitive reward–related neuronal structures involved in the anticipation of pleasure (Dichter et al., 2009).

Behavioral models suggest that BA increases positively reinforcing experiences from engagement in rewarding activities (Hopko et al., 2003). Some authors have recently emphasized the importance of further investigating the role of the appetitive reward system in BA models (Blairy et al., 2020; Forbes, 2020; Nagy et al., 2020). The appetitive reward system is associated with two distinct temporal orientations of pleasure. The first one involves savoring future positive events, also called anticipatory pleasure or *wanting*, while the second involves savoring present events, also called consummatory pleasure or *liking* (Berridge and Kringelbach, 2008; Admon and Pizzagalli, 2015). Both components have been reported to be disturbed in depression (Treadway and Zald, 2011; Wu et al., 2017). However, in a subclinically depressed sample, anticipatory pleasure was identified as a significant predictor of subsequent consummatory pleasure, suggesting that, in depression, the wanting component is more clinically relevant than the liking component (Li et al., 2019). Additionally, lack of anticipatory pleasure is predictive of a poorer course of depression (Morris et al., 2009) and suicidality (Winer et al., 2014). Regarding the interplay between anticipatory pleasure and activation, previous research suggests two hypotheses. First, anticipatory pleasure might affect depressed individuals’ motivation to engage in potentially rewarding experiences. Empirical data support this hypothesis, as previous studies reported that anticipatory pleasure was a significant predictor of motivation to exert effort for rewards in non-depressed (Geaney et al., 2015) and depressed samples (Sherdell et al., 2012). Second, anticipatory pleasure might be influenced by engagement in rewarding experiences. Indeed, Beevers and Meyer (2002) reported that rewarding experiences predict positive expectations, which then significantly predict symptoms of depression. Furthermore, Bakker et al. (2017) reported that active behaviors influenced reward anticipation in a subclinically depressed sample. Given previous findings suggesting that activation, behavioral avoidance, anticipatory pleasure, and brooding predict depressive symptoms, the present study sought to examine the relationships between these processes and depressive symptoms, as well as between activation and anticipatory pleasure.

Even though BA is associated with medium to large effect sizes in the reduction of depressive symptomatology, its efficacy could still be enhanced (Cuijpers et al., 2020). A promising way to enhance the efficacy of BA is to combine it with another therapeutic intervention (Averill et al., 2019; Van den Bergh et al., 2020). Cognitive Control Training (CCT) is a recent empirically validated cognitive treatment of depression, which activates prefrontal neural networks with repeated cognitive exercises designed to engage those structures (Koster et al., 2017). CCT uses working memory tasks to strengthen prefrontal neural activation (Koster et al., 2017). It aims to increase CC abilities in order to reduce cognitive biases (i.e., attentional biases- AB_s_) and non-adaptive cognitive regulation strategies (e.g., brooding) and consequently reduce depression (De Raedt and Koster, 2010; Koster et al., 2011). As the impaired disengagement hypothesis posits, low CC resources lead to generally impaired attentional disengagement (Koster et al., 2011). This impaired disengagement maintains AB_s_ (i.e., disengagement from sad cues and from happy cues) and brooding, which are two vulnerability factors for depression [for a review, see LeMoult and Gotlib, (2019)]. Indeed, empirical data suggest that CCT may reduce depressive symptoms and brooding in depressed patients treated with a CCT (Siegle et al., 2014; Vanderhasselt et al., 2015). In addition, previous studies have indicated that CC’s influence on depressive symptoms might be at least partly mediated by brooding (Hsu et al., 2015; Hoorelbeke and Koster, 2017), as well as the impact of AB_s_ on depressive symptoms (Sanchez et al., 2019; Yaroslavsky et al., 2019). Given previous findings suggesting that CC and AB_s_ predict brooding and depressive symptoms, this study examined the relationships between these processes and depressive symptoms, as well as among the different processes.

In light previous results, the combination of BA and CCT could amplify the efficacy of BA because the two treatments act on different depressive vulnerability processes (activation, behavioral avoidance, and anticipatory pleasure for BA; CC and AB_s_ for CCT), as well as on a common process, namely brooding. Indeed, if cognitive resources influence brooding, it is possible that the combination of BA and CCT could strengthen individual capacities to disengage from brooding in the long term. To date, one study has investigated the combination of a CCT and a BA treatment in a clinically depressed sample (Moshier and Otto, 2017). Both conditions (BA in adjunction to CCT and BA in adjunction to a sham procedure) were associated with a substantial reduction in depressive symptoms and brooding. However, repeated measures ANOVAs used to examine symptoms as functions of the interaction between time and treatment condition were non-significant and all effect sizes were small (all ηp^2^ < 0.07). The absence of the expected significance could be attributable to the small sample size (*n* = 34), which also hindered the identification of potential mediators of treatment effects, including brooding.

**Figure 1:**
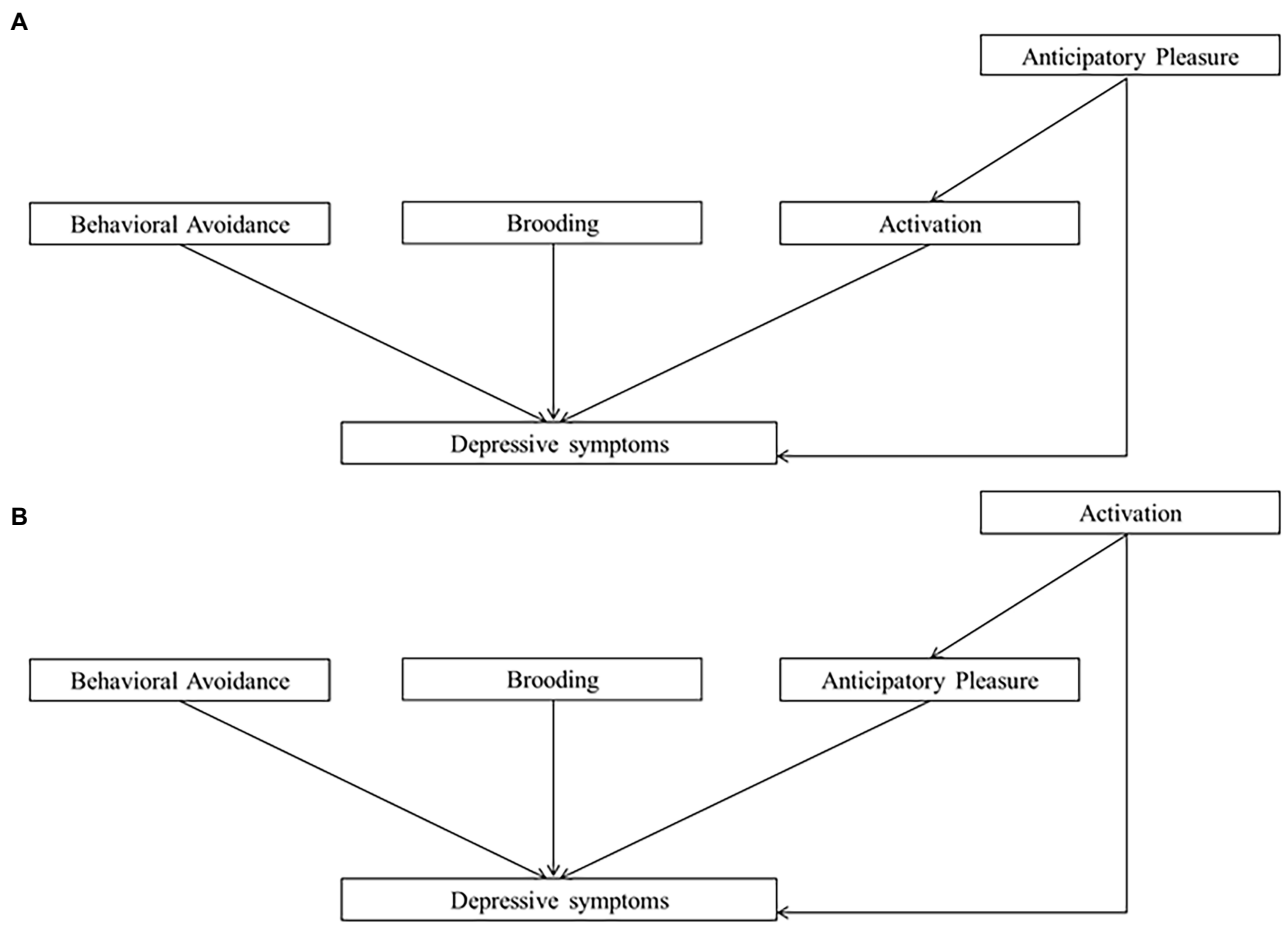
Behavioral models. **(A)** Model 1 (behavioral model 1). **(B)** Model 2 (behavioral model 2).

**Figure 2:**
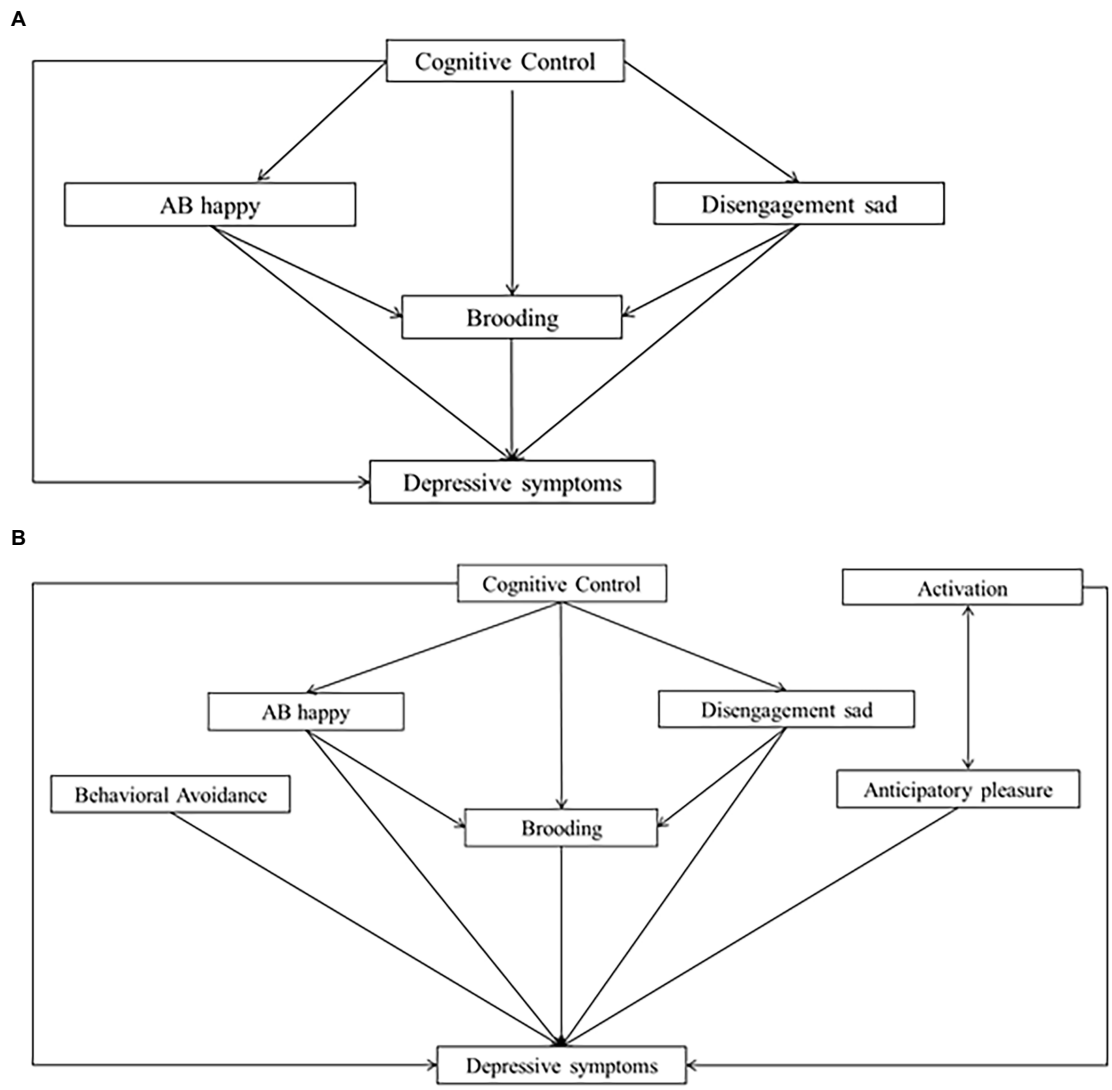
Cognitive and comprehensive models. **(A)** Model 3 (cognitive model). **(B)** Model 4 (comprehensive model).

Using path analysis models and a large sample of participants, we sought to investigate whether the adjunction of certain cognitive processes to BA processes could predict more depressive symptoms. If this is the case, adding cognitive training to BA could make the treatment more efficient (i.e., reduce depressive symptoms). This study investigates relationships between depressive symptoms and, on the one hand, the target processes of BA treatment (activation, behavioral avoidance, anticipatory pleasure and brooding), and on the other hand, the target processes of CCT (CC, AB_s_, and brooding). Overall, four models were tested. First, we tested the relevance of two behavioral models with Activation, Behavioral Avoidance, Brooding and Anticipatory Pleasure as processes predicting depressive symptoms. Model 1 tested the hypothesis that Anticipatory Pleasure would partially predict Activation, which in turn would predict depressive symptoms. Model 2 tested the reverse hypothesis: that activation would partially predict Anticipatory Pleasure, which in turn would predict depressive symptoms. Second, we tested a cognitive model (Model 3) with CC, AB_s_ and Brooding as predictors of depressive symptoms. In Model 3, we also tested the hypothesis that CC would partially predict Brooding, which in turn would predict depressive symptoms, and that AB_s_ would partially predict Brooding, which in turn would predict depression symptoms. Third, a comprehensive model was tested (Model 4) with the hypothesis that the integration of behavioral and cognitive processes would explain more of the variance in brooding and depressive symptoms than each model separately. Schemas depicting the four models are presented in Figures 1 and 2. Finally, we identify the unique variance in depressive symptoms that is explained by behavioral and cognitive processes, independent of the other variables. This analysis helps to investigate the most promising therapeutic targets and isolate the most relevant therapeutic levers.

## Materials and Methods

### Participants

The participants were 549 unselected French-speaking adults aged from 18 to 64 years. Advertisements, university intranets, and the waiting rooms of healthcare centers were used to recruit participants. Data analyses were based on 520 adults (338 females, 182 males) with a mean age of 30.99 years (SD = 11.89; range: 18–64). Five participants were excluded because of a history of psychotic mental disorders, two were excluded because of a history of substance abuse or dependence including alcohol (less than 3 years of abstinence—except nicotine or caffeine), 11 for a history of neurological disorder, and two for the use of anxiolytics or other drugs on the day of the assessment. In addition, two participants were excluded because of anti-psychotic medication, two because of recent changes in antidepressant medication (less than 4 weeks). Participants had normal or corrected vision and no history of bipolar disorder. Five participants were also excluded from the analysis due to extensive missing data.

### *A Priori* Power Analysis

For path analysis, a sample size of more than 500 participants is considered as very good to test confirmatory models (Comfrey and Lee, 1992). However, some authors suggest that these rules are problematic because they are not model-specific and may lead to grossly over-or underestimated sample size requirements (Wolf et al., 2013). Therefore, we have estimated the optimal sample considering the expected effect size (RMSEA <0.05), type of model (path analysis), degrees of freedom based on the number of parameters estimated by the model (df = 12) to reach a statistical power of 0.80. The analysis revealed that the optimal sample size for the comprehensive model (Model 4) is 578 participants.

### Materials

The cross-sectional study was conducted before the COVID-19 lockdown. The materials consisted of a computerized task and self-report questionnaires.

#### Demographic Questionnaire

A sociodemographic questionnaire addressed questions about age, gender, marital status, employment status, medication, quality of vision, neurological history, and past depressive episodes.

#### Depressive Symptomatology

The Beck Depression Inventory—Second Edition (BDI-II) is a 21-item scale that assesses the severity of depressive symptoms in the previous 2 weeks (Beck et al., 1996). Higher scores indicate greater severity. We used the validated French version of the scale (Centre de Psychologie appliquée, 1996). In the present sample, Cronbach’s *α* for the whole scale was 0.86.

#### Activation and Behavioral Avoidance

The Behavioral Activation for Depression Scale—Short Form (BADS-SF) is a 9-item scale assessing behavioral activation (Manos et al., 2011). Two subscales are identified: Activation (four items) and Avoidance (three items). We removed one item from the Avoidance subscale (item 7), which refers to brooding, to avoid a conceptual overlap between behavioral avoidance and brooding. Higher scores indicate higher behavioral activation and behavioral avoidance levels, respectively. We used the validated French version of the scale (Wagener et al., 2015). In our sample, Cronbach’s *α* was 0.77 for the activation subscale and 0.68 for the behavioral avoidance subscale.

#### Anticipatory Pleasure

The Savoring Belief Inventory (SBI) is a 24-item scale assessing individuals’ attitudes regarding savoring positive experiences (Bryant, 2003). Three subscales are identified, one related to pleasure in reminiscence of past events, one related to pleasure in relation to the present moment, and one related to pleasure in anticipation of future events, each represented by eight items. We used only the last subscale to measure anticipatory pleasure. The score is calculated by subtracting the sum score of the negatively phrased items from the sum score of positively phrased items. Higher scores indicate a higher level of savoring of pleasant events. We used the validated French version of the scale (Golay et al., 2018). In our sample, Cronbach’s *α* was 0.77 for positive anticipation and 0.67 for negative anticipation.

#### Brooding

The Ruminative Response Scale (RRS) is a 22-item scale assessing rumination when respondents feel depressed, sad or discouraged (Treynor et al., 2003). Two subscales are identified, one related to brooding (five items) and one related to reflection. The reflection subscale was not reported because this aspect of rumination is more adaptive than brooding and less related to depression. Higher scores on the brooding subscale indicate a higher level of brooding. We used the validated French version of the scale.^1^ In this sample, Cronbach’s *α* for the brooding subscale was 0.72.

#### Disengagement From Sad Cues and Attention to Happy Cues

The exogenous cueing task (ECT) is a reaction-time-based attention task, which was programmed using OpenSesame software and was run on a computer with a 60 Hz, 15-inch color monitor. The original exogenous cueing task asked participants to detect a visual target presented in the left or right peripheral location of the screen (Posner, 1980). In affective science, the paradigm has been modified by using emotional and neutral cues to allow a comparison of their attentional processing.

**Figure 3:**
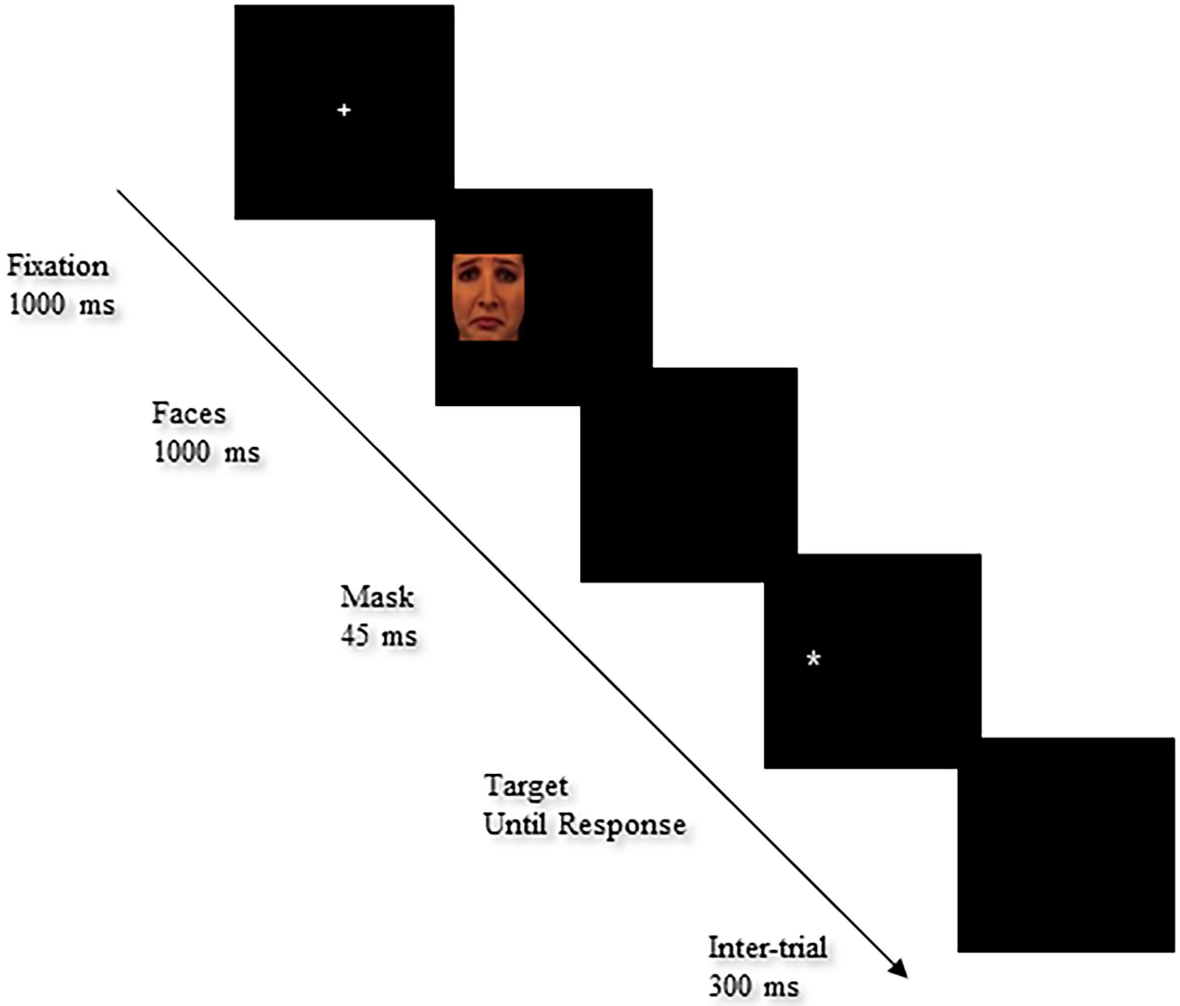
Sequence of events in the detection cueing task. Facial image reproduced from Karolinska Directed Emotional Face (KDEF; Lundqvist et al., 1998; AF13SAS).

The task was created with faces (14 happy, 14 sad and 14 neutral) selected from the Karolinska Directed Emotional Face (KDEF) database (Lundqvist et al., 1998; Goeleven et al., 2008). Faces were sized 280 pixels high X 280 pixels wide with visual angles of 5.81°×5.81°. Each trial started with the presentation of a fixation cross for 1,000 ms in the center of the screen. Then, the emotional cue was presented on the left or right side of the screen for 1,000 ms followed by a “mask” screen for 45 ms. Finally, the target (“*”) was presented until a response was made. A black background intertrial was then presented for 300 ms before the next trial started. The sequence of events in a test trial is depicted in Figure 3. In the test trials, half of positive faces were valid (28 trials; left cue–left target and right cue–right target) and half were invalid (28 trials). The same proportions were used for sad and neutral faces, with half valid (28 trials) and half invalid (28 trials; left cue–right target and right cue–left target). Fifty-six of the remaining stimuli were no-cue and 10 were digital trials to enhance the probability that participants would maintain their gaze in the middle of the screen. The fixation cross was replaced by a digit for 450 ms, after which no cue or target followed and participants were instructed to report the digit aloud as quickly as possible. The stimuli were presented at random in the left or right hemifield with an equal number of presentations for each stimulus (twice) and each emotional category (sad, happy, and neutral; 56 trials each).

For sad faces, an attentional disengagement score was calculated by subtracting the mean reaction times (RTs) of invalid neutral trials from the mean RTs of invalid emotional trials (Koster et al., 2005). For happy faces, we subtracted the mean RTs for valid trials from the mean RTs for invalid trials in order to compute a cue validity (CV) index. Although preliminary research data suggest that depressed participants engage more slowly with positive stimuli than non-depressed participants (Sanchez et al., 2017), the nature of the attentional components involved in bias related to positive cues remains poorly understood. Then, we computed CV scores to measure attentional bias to positive cues to include both attentional components. With longer stimulus-onset asynchronies, as were used in this study, a positive cue validity effect suggests that attention is maintained on the cue.

The split-half reliability indices were computed separately for each task *via* Spearman-Brown correlations with the first and second half of trials in each experimental condition. Spearman-Brown correlations ranged from 0.43 (Happy valid) to 0.48 (Happy invalid). We ran the same analyses for CV scores for happy cues and disengagement scores from sad cues. The Spearman-Brown correlations computed were 0.006 for disengagement from sad cues and 0.048 for CV for happy cues.

#### Cognitive Control

A computerized version of the Paced Auditory Serial-Addition Task (PASAT) was used to measure participants’ updating abilities and monitoring of representations within working memory, one aspect of CC (Gronwall, 1977; Tombaugh, 2006). In the task, 60 numbers (from 1 to 9) are presented successively. Subjects were asked to add each number to the one that immediately preceded it, which interferes with the updating of the last heard digits in working memory. The task is divided into four trials that differ in terms of the speed with which the numbers are presented (one number every 2.4, 2.0, 1.6, or 1.2 s). The outcome measures were the number of correct responses for each of the four experimental trials. The total accuracy score served as a behavioral indicator of CC. The split-half reliability of this measure (Spearman-Brown corrected) computed with the first two series and the last two series of trials was 0.89.

### Procedure

The evaluations were administered individually in a quiet room with dim light. Participants first completed the computerized tasks and then the self-report questionnaires. Participants started with the two computerized tasks, introduced in a counterbalanced order. The questionnaires were administered in the same order for all participants. To complete the spatial cueing tasks, participants were seated 60 cm from the computer screen. They were asked to detect, as quickly as possible, the location of the target (“*”)—left mouse button with left index finger; left side; right mouse button with right index finger, right side—without sacrificing accuracy. The instructions were presented on screen. Participants were informed that a cue would precede the presentation of the target and that the cue correctly predicted the location of the target in some but not all trials. Participants practiced the attentional task for 15 trials. The test phase consisted of one block with 234 trials. We presented the trials in a new random order for each participant. The total time for data acquisition was approximately 1 h (i.e., preparation of the participant, familiarization with the tasks, breaks, and debriefing).

The local Ethics Committee approved the study.^2^ All participants gave their written informed consent.

### Data Preparation

First, we discarded trials with errors from the analyses (0.007% of all data). To take each participant’s processing speed into account, we followed Ratcliff's (1993) guidelines for dealing with outliers. To do so, we decided to rely on an individual approach based on deviations below or above each participant’s mean for each experimental condition. Participants’ RTs more than three standard deviations from their individual mean RT for all indices (Invalid Sad, Valid Sad, Invalid Happy, Valid Happy, Invalid Neutral, and Valid Neutral) were considered as outliers. These outlying RTs were excluded on the basis that they indicated anticipatory responses (0.001% of all data) or delayed responses (0.01% of all data). None of the participants exhibited more than 10% of erroneous response or outliers. We conducted the analyses on the remaining 99.98% of the data.

### Statistical Analysis

The Shapiro–Wilk test suggested that all variables were non-normally distributed (all *p_s_* < 0.002). First, we computed non-parametric Spearman correlations between all variables to describe our group characteristics using JASP Version 0.13.1 (JASP Team, 2020).

Thereafter, path analysis with a maximum-likelihood estimation method was computed with the Lavaan package in R, version 0.6–8 (Rosseel, 2012). According to Rosseel, the parameters estimated by this method are consistent with non-normal data.^3^ The goodness of fit is indicated by a non-significant *χ*^2^. If the chi-square is significant, a *χ*^2^/degrees of freedom ratio of less than 2 indicates a good fit, while a result of less than 3 is acceptable (Cangur and Ercan, 2015). We also computed several other fit statistics, including the Root Mean Square Error of Approximation (RMSEA), the Standardized Root Mean square Residual (SRMR), the Tucker-Lewis Index (TLI), and the Comparative Fit Index (CFI; Bentler, 1990; Hu and Bentler, 1999; Cangur and Ercan, 2015). An RMSEA between 0.05 and 0.08, SRMR <0.10, TLI > 0.95, and CFI > 0.95 are generally interpreted as indicating an acceptable fit (Bentler, 1990; Schermelleh-Engel et al., 2003). To compare Models 1 and 2, we computed two additional indices: Akaike’s Information Criterion (AIC) and Sample-size adjusted Bayesian Information Criterion (BIC). Lower AIC and BIC scores indicate a better model fit (Akaike, 1973). Standardized path coefficients are reported in each figure (Wright, 1934). To control for measurement error, we conducted an additional bootstrap on standard errors by randomly resampling the data 10,000 times.

In order to more directly test the influence of behavioral processes (Activation, Behavioral Avoidance, Anticipatory Pleasure), cognitive processes (CC, AB_s_) and the common process (Brooding) on depressive symptoms, seven hierarchical linear regression analyses were performed on depressive symptoms to measure the variance in depressive symptoms that is explained by each predictor separately after controlling for the effect of the others. Hierarchical regression model analyses were done with JASP Version 0.13.1 (JASP Team, 2020).

Following recommendations on research transparency and replicability, the OpenSesame version of the task, the stimuli, and the de-identified data can be freely downloaded *via* the following link: https://osf.io/hfj8a/.

## Results

### Group Characteristics

The full sample had a mean BDI-II (Beck et al., 1996) score of 10.02 (SD = 7.36, range 0–48). Their demographic characteristics appear in Table 1 and the means and standard deviations of all measures are shown in Table 2. Table 3 presents Spearman non-parametric correlations between all variables. Most correlations were statistically significant except the correlations including disengagement from sad cues and attentional bias to happy cues (ranging from *r* = 0.00 to *r* = −0.07, all *p_s_* > 0.05). In addition, non-significant correlations were reported between CC and brooding (*r* = −0.06, *p* > 0.05), and CC and behavioral avoidance (*r* = −0.05, *p* > 0.05). The strongest correlations were found between brooding and depression (*r* = 0.50, *p* < 0.001), behavioral avoidance and depression (*r* = 0.46, *p* < 0.001), activation and depression (*r* = −0.41, *p* < 0.001) and brooding and behavioral avoidance (*r* = 0.32, *p* < 0.001).

**Table 1:** Group characteristics.

Measure	
*N*	520
Age	31 (11.89)
Gender (M/F)	182/338
Education level (number of years successfully completed)	14.12 (2.26)
Origin
Caucasian	87.31%
African	12.31%
Asian	0.38%
Employment status
Student	37.50%
Laborer	8.27%
Employee	35.96%
Executive	4.81%
Self-employed	6.15%
Homemaker	1.15%
Unemployed	5.38%
Retired	0.19%
Missing data	0.58%
Unable to work	1.54%
Marital status
Single	69.23%
Married	17.88%
Legally cohabiting	5.77%
Widowed	0.39%
Divorced	6.73%
Other	0%
Live in a couple	60.96%
Have children	33.08%
Report at least one past depressive episode with medical treatment	24.42%
Current depressive episode	5%
Currently on psychotropic medication (antidepressant)	2.12%
SSRI	8/11
SNRI	3/11
Currently on psychotropic medication (anxiolytic)	0.77%

Standard deviations are shown in parentheses. SSRI, selective serotonin reuptake inhibitor. SNRI, selective norepinephrine reuptake inhibitor.

**Table 2:** Means and standard deviations for all variables.

Measure	Range (min-max)	Mean (SD)
Depressive symptoms (BDI-II)	0–63	10.02 (7.36)
Activation (BADS-SF)	0–24	13.40 (4.76)
Behavioral avoidance (BADS-SF)	0–12	4.23 (4.30)
Anticipatory pleasure (SBI)	−24–24	12.15 (7.52)
Brooding (RRS)	5–20	10.79 (3.30)
Disengagement from sad cues	–	0.08 (27.02)
CV for happy cues	–	−22.79 (42.55)
Cognitive control (PASAT)	0–60	50.83 (9.02)

SD, standard deviation; BDI-II, beck depression inventory-II; BADS-SF, behavioral activation for depression scale—short form; SBI, savoring belief inventory; RRS, ruminative response scale; CV, cue validity; PASAT, paced auditory serial-addition task.

**Table 3:** Spearman’s correlations between all variables.

Measures	Activation	Behav. avoidance	Ant. pleasure	Brooding	CV happy	Dis. sad	CC
Activation	–						
Behav. avoidance	−0.23^***^	–					
Ant. pleasure	0.26^***^	−0.13^**^	–				
Brooding	−0.27^***^	0.32^***^	−0.11^*^	–			
CV happy	−0.10^*^	0.11^*^	−0.06	0.10^*^	–		
Dis. sad	0.02	−0.01	−0.003	−0.07	0.00	–	
CC	0.12^**^	−0.05	0.07	−0.06	−0.04	0.02	–
Depressive sympt.	−0.41^***^	0.46^***^	−0.26^***^	0.50^***^	0.04	−0.03	−0.14^**^

*** *p* < 0.001;

** *p* < 0.01;

* *p* < 0.05.

Behav. avoidance, behavioral avoidance; Ant. pleasure, anticipatory pleasure; CV happy, cue validity for happy cues; Dis. sad, disengagement from sad cues; CC, cognitive control; Depressive sympt., depressive symptoms.

### Path Analysis Models

We defined our path analysis models based on literature and theoretical frameworks. First, we tested a behavioral model in which activation, behavioral avoidance, brooding and anticipatory pleasure were defined as predictors of depressive symptoms and anticipatory pleasure was defined as an additional predictor of activation (Model 1). To explore an alternative relationship between anticipatory pleasure and activation, we tested Model 2, in which activation, behavioral avoidance, brooding and anticipatory pleasure were defined as predictors of depressive symptoms and activation was defined as an additional predictor of anticipatory pleasure. In Models 1 and 2, covariances were indicated between activation, behavioral avoidance and brooding. In the cognitive model (Model 3), we defined CC, the two kinds of AB_s_ and Brooding as predictors of depressive symptoms, CC as a predictor of the two AB_s_ and brooding, and the two AB_s_ as additional predictors of brooding. Finally, Model 4 tested a comprehensive model comprising a combination of the best behavioral model (Model 1 or Model 2) and Model 3.

The statistics for Model 1 did not suggest an adequate fit for the data (*χ*(2)^2^ = 14.19 *p* < 0.001, *χ*^2^/df = 7.10, RMSEA of 0.108, SRMR = 0.058, TLI = 0.862, CFI = 0.972, AIC = 15188.482, and BIC = 15202.517). However, all predictors of depressive symptoms were statistically significant (*β* = −0.36 for activation, *β* = 0.68 for behavioral avoidance*, β* = 0.74 for brooding, and *β* = −0.14 for anticipatory pleasure, all *p_s_* < 0.001). Anticipatory pleasure was a significant predictor of activation (*β* = 0.13, *p* < 0.001) and each covariance was significant (all *p_s_* < 0.001). The model explained 42% of the variance in depressive symptoms and 4% of the variance in activation.

**Figure 4:**
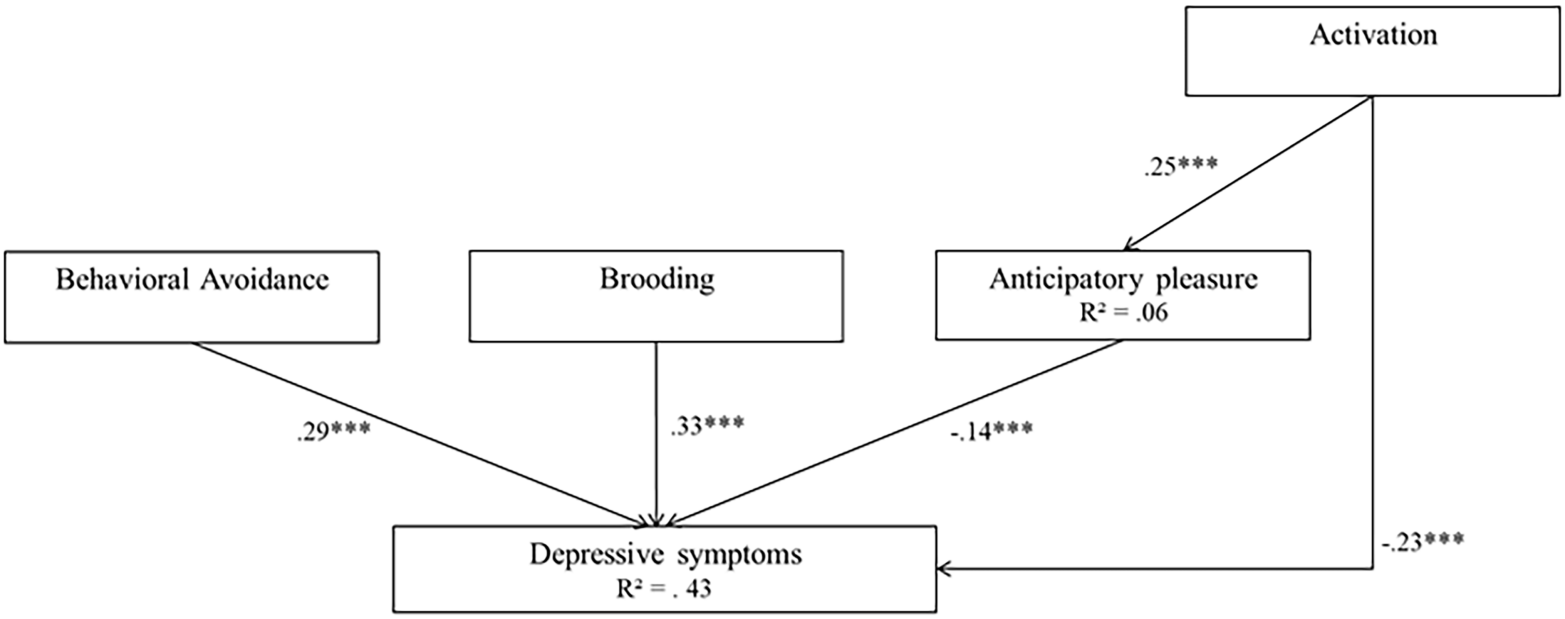
Model 2 (behavioral model 2). Initial structural equation model. Rectangles represent observed measured variables. Values are path-standardized coefficients. *R*^2^ represents the proportion of the variance for a dependent variable that is explained by an independent variable or variables in a regression model. ^***^indicates a significant path coefficient at *p* < 0.01. ^*^indicates a significant path coefficient at *p* < 0.05. (black: *p* < 0.05; gray: *p* > 0.05).

Model 2 was associated with high goodness-of-fit indices (*χ*(2)^2^ = 4.41 *p* = 0.11, RMSEA of 0.048, SRMR = 0.028, TLI = 0.973, CFI = 0.995, AIC = 15178.712, and BIC = 15192.747). In this model, activation, behavioral avoidance, brooding and anticipatory pleasure were significant predictors of depressive symptoms (*β* = −0.36 for activation, *β* = 0.68 for behavioral avoidance, *β* = 0.74 for brooding, and *β* = −0.14 for anticipatory pleasure, all *p_s_* < 0.001). In addition, activation was a significant predictor of anticipatory pleasure (*β* = 0.39, *p* < 0.001). The model explained 43% of the variance in depressive symptoms, and 6% of the variance in anticipatory pleasure. In both models, all path coefficient signs corroborated the expectations. AIC and BIC indices were lower in Model 2 than in Model 1, suggesting that Model 2 fit the data well. Model 2 is represented in Figure 4.

**Figure 5:**
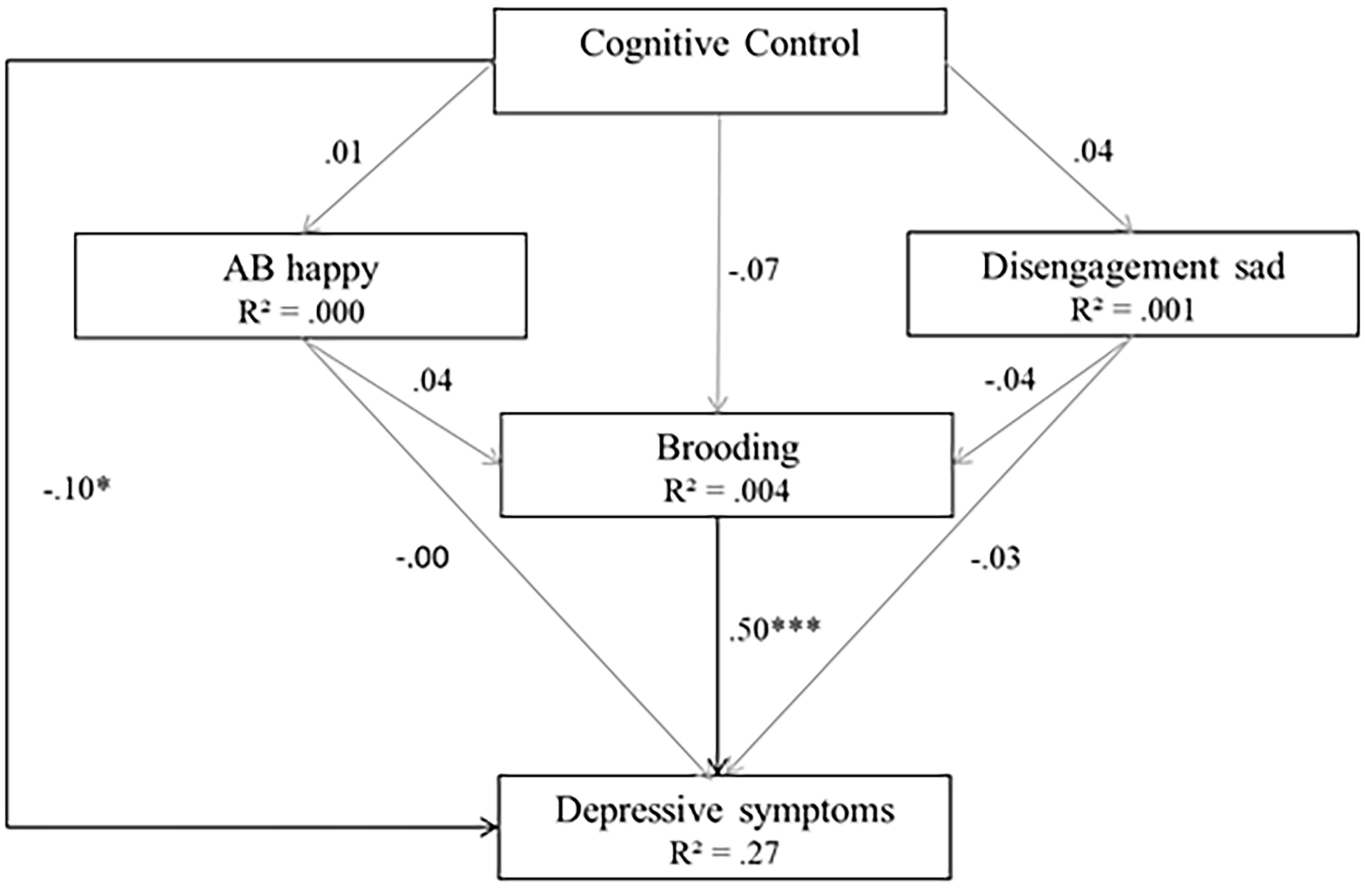
Model 3 (cognitive model). Initial structural equation model. Rectangles represent observed measured variables. Values are path-standardized coefficients. *R*^2^ represents the proportion of the variance for a dependent variable that is explained by an independent variable or variables in a regression model. ^***^indicates a significant path coefficient at *p* < 0.01. ^*^indicates a significant path-coefficient at *p* < 0.05. (black: *p* < 0.05; gray: *p* > 0.05).

High goodness-of-fit indices were associated with the cognitive model (Model 3; *χ*(1)^2^ = 0.081, *p* = 0.776, RMSEA <0.001, SRMR = 0.003, TLI = 1.00, CFI = 1.00). However, not all hypothesized predictors of depressive symptoms were statistically significant. As expected, brooding and CC were significant predictors of depressive symptoms (*β* = 1.11, *p* < 0.001 for brooding, *β* = −0.80, *p* < 0.05 for CC). However, non-significant regressions were reported between the two AB_s_ and depressive symptoms (*β*_s_ = 0.00, *p*_s_ > 0.05), between CC and the two AB_s_ (*β* = 0.19 for happy cues and *β* = 0.64 for sad cues, *p_s_* > 0.05) and between CC and brooding (*β* = −0.03, *p* > 0.05). The model explained 26% of the variance in depressive symptoms and less than 1% of the variance in Brooding (*R*^2^ = 0.004) and AB_s_ (*R*^2^ = 0.00 for happy cues, *R*^2^ = 0.001 for sad cues). Model 3 is depicted in Figure 5.

A majority of path coefficient signs corroborated expectations except the signs between CC and disengagement from sad cues and between attention to happy cues and brooding. Furthermore, the path signs between disengagement from sad cues and brooding, as well as between disengagement from sad cues and depressive symptoms, were unexpected.

**Figure 6:**
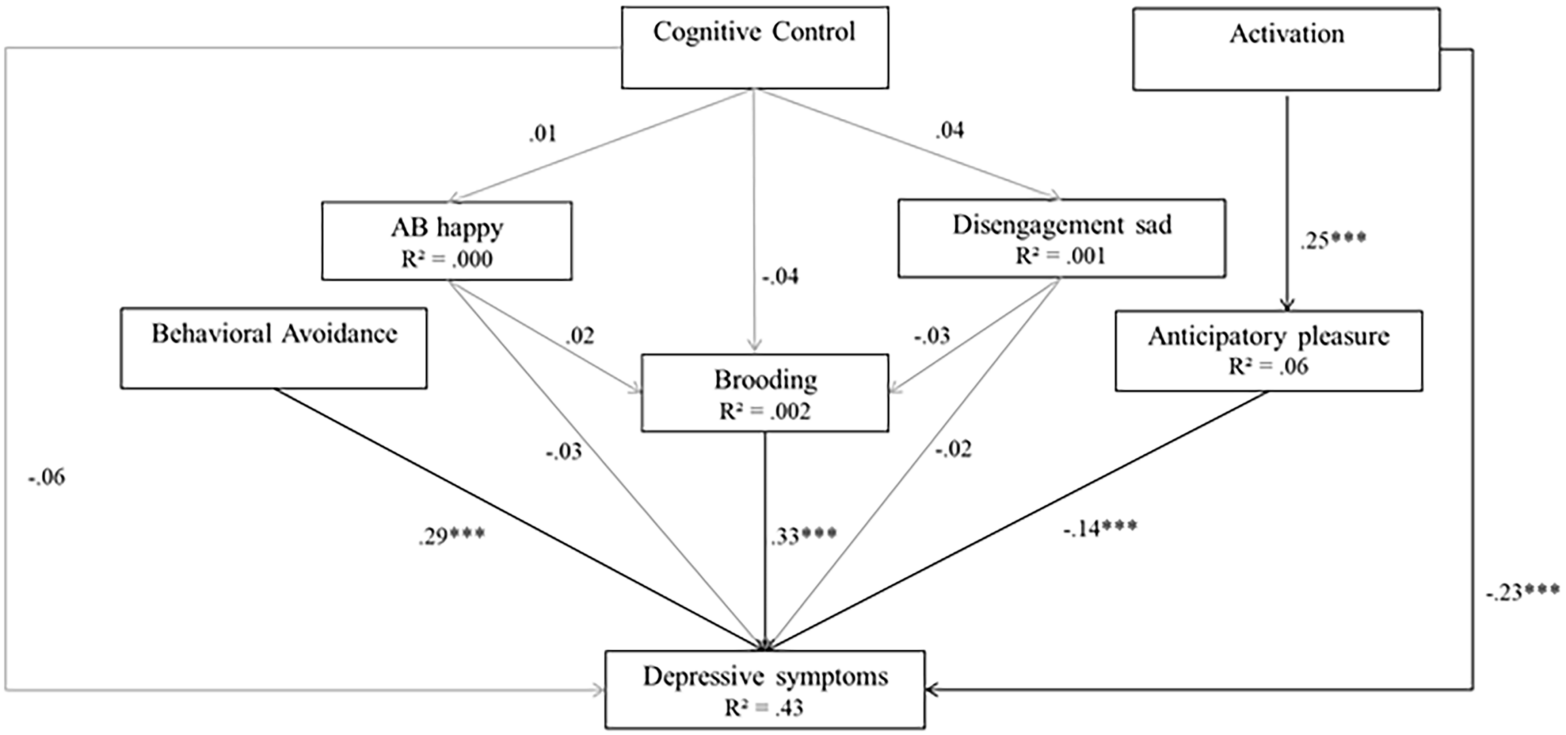
Model 4 (comprehensive model). Initial structural equation model. Rectangles represent observed measured variables. Values are path-standardized coefficients. *R*^2^ represents the proportion of the variance for a dependent variable that is explained by an independent variable or variables in a regression model. ^***^indicates a significant path coefficient at *p* < 0.01. ^*^indicates a significant path-coefficient at *p* < 0.05. (black: *p* < 0.05; gray: *p* > 0.05).

Finally, Model 4 tested a comprehensive model (incorporating Model 2 and Model 3). Model 4 produces high goodness-of-fit indices (*χ*(12)^2^ = 18.98, *p* = 0.09, RMSEA of 0.033, SRMR = 0.040, TLI = 0.963, CFI = 0.984). In Model 4, activation, behavioral avoidance, brooding, and anticipatory pleasure were significant predictors of depressive symptoms (*β* = −0.35 for activation; *β* = 0.68 for behavioral avoidance; *β* = 0.73 for brooding, and *β* = −0.14, for anticipatory pleasure, all *p_s_* < 0.001). However, the two AB_s_ and CC did not significantly predict depressive symptoms (*β* = −0.002 for happy cues, *β* = −0.001 for sad cues, and *β* = −0.05 for CC, all *p*_s_ > 0.05). In addition, CC did not significantly predict the AB_s_ (*β* = 0.19 for happy cues and 0.64 for sad cues, *p* > 0.05) or brooding (*β* = −0.01, *p* > 0.05), and the AB_s_ did not significantly predict brooding (*β* = −0.000 for happy cues, and *β* = −0.001 for sad cues, all *p_s_* > 0.05). Finally, activation was a significant predictor of anticipatory pleasure (*β* = 0.39, *p* < 0.001). Model 4 explained 43% of the variance in depressive symptoms, 6% of the variance in anticipatory pleasure, and less than 1% of the variance in brooding and AB_s_. Figure 6 presents Model 4.

### Complementary Analyses

Bootstrap analysis conducted on standard errors generated similar standard errors and *Z* values than previous results and lead to the same conclusions. Four supplemental tables reporting the estimate, standard error, *Z* value with and without bootstraps, and path-standardized coefficients can be downloaded *via* the following link: https://osf.io/hfj8a/.

Because the comprehensive model shows a large number of statistically insignificant paths, making this model more complex than necessary, we computed additional analysis testing a comprehensive model where ABs were removed considering the high standard error for the ABs variances and insignificant paths between ABs and brooding, and ABs and depressive symptoms. The analyses generated similar results with similar conclusions with still non-significant paths between CC and depressive symptoms and CC and brooding. The simplified Model 4 tested produce acceptable fit indices (*χ*(5)^2^ = 14.513 *p* = 0.013, *χ*^2^/df = 2.90, RMSEA of 0.060, SRMR = 0.046, TLI = 0.937, CFI = 0.979). In this simplified comprehensive model, activation, behavioral avoidance, brooding, and anticipatory pleasure were significant predictors of depressive symptoms (*β* = −0.35 for activation; *β* = 0.68 for behavioral avoidance; *β* = 0.73 for brooding, and *β* = −0.13 for anticipatory pleasure, all *p*_s_ < 0.001). However, CC did not significantly predict depressive symptoms (*β* = −0.05 *p* > 0.05). In addition, CC did not significantly predict brooding (*β* = −0.01, *p* > 0.05). Finally, activation was a significant predictor of anticipatory pleasure (*β* = 0.38, *p* < 0.001). This simplified model explained 43% of the variance in depressive symptoms, 6% of the variance in anticipatory pleasure, and less than 1% of the variance in brooding.

### Hierarchical Regression Analyses

We computed hierarchical regression models to measure the unique variance in depressive symptoms that might be explained by each predictor after controlling for the effects of the other predictors. Step 1 provides simple linear regressions assessing the amount of variance that could be attributed to each predictor. In order to verify whether the contribution of one predictor might be reduced to a non-significant account after controlling for all other predictors, step 2 includes the six remaining processes as predictors. We also computed hierarchical regression models to measure the variance in brooding that might be explained by CC and AB_s_ after controlling for the effects of the others. We compared predictors for statistically significant changes in the explained variance *R*^2^ by computing a partial *F*-statistic. The results are summarized in Table 4.

**Table 4:** Hierarchical linear regressions of depressive symptoms.

Step	Predictors	*R* ^2^	Adjusted *R*^2^	Δ*R*^2^	Δ*F*	Value of *p*
1	Activation	0.185	0.183	0.183	117.46	< 0.001
1	Behav. avoid. & Ant. pleas. & Brood. & CV H & Dis. sad & CC	0.402	0.395	0.402	57.36	< 0.001
2	Activation	0.446	0.439	0.045	41.494	< 0.001
1	Behavioral avoidance	0.216	0.214	0.216	142.49	< 0.001
1	Act. & Ant. pleas. & Brood. & CV H & Dis. sad & CC	0.372	0.365	0.372	50.64	< 0.001
2	Behavioral avoidance	0.446	0.439	0.074	68.81	< 0.001
1	Brooding	0.258	0.256	0.258	179.80	< 0.001
1	Act. & Behav. avoid. & Ant. pleas. & CV H & Dis. sad & CC	0.356	0.349	0.356	47.35	< 0.001
2	Brooding	0.446	0.439	0.090	83.21	< 0.001
1	Anticipatory pleasure	0.078	0.076	0.094	43.942	< 0.001
1	Act. & Behav. avoid. & Brood. & CV H & Dis. sad & CC	0.429	0.422	0.429	64.28	< 0.001
2	Anticipatory pleasure	0.446	0.439	0.017	15.94	< 0.001
1	CV happy	0.000	−0.002	0.000	0.192	0.66
1	Act. & Behav. avoid. & Ant. pleas. & Brood. & Dis. sad & CC	0.444	0.437	0.444	68.18	< 0.001
2	CV happy	0.446	0.439	0.003	2.519	0.11
1	Disengagement sad	0.002	−0.000	0.002	0.875	0.35
1	Act. & Behav. avoid. & Ant. pleas. & Brood. & CV H & CC	0.446	0.440	0.446	68.91	< 0.001
2	Disengagement sad	0.446	0.439	0.000	0.093	0.76
1	CC	0.018	0.017	0.018	9.73	0.00
1	Act. & Behav. avoid. & Ant. pleas. & Brood. & CV H & Dis. sad	0.443	0.437	0.443	68.03	< 0.001
2	CC	0.446	0.439	0.003	3.029	0.08

Act., activation; Behav. avoid., behavioral avoidance; Ant. pleas., anticipatory pleasure; CV H, cue validity for happy cues; Dis. sad, disengagement from sad cues; CC, cognitive control.

The Step 1 models revealed that activation, behavioral avoidance, anticipatory pleasure, brooding and CC explained a significant proportion of the variance in depressive symptoms (18.3% for activation, 21.4% for behavioral avoidance, 25.6% for brooding, 9.3% for anticipatory pleasure, and 1.7% for CC). AB_s_ were not significant predictors of the variance in depressive symptoms, with less than 1% of the variance in depressive symptoms explained by the two AB_s_ (*R*^2^ < 0.01). The Step 2 models revealed that activation, behavioral avoidance, anticipatory pleasure, and brooding were still significant predictors of depressive symptoms after controlling for the influence of other predictors (Δ*R*^2^ = 0.045 for activation, Δ*R*^2^ = 0.074 for behavioral avoidance, Δ*R*^2^ = 0.090 for brooding, Δ*R*^2^ = 0.017 for anticipatory pleasure). However, the variance in depressive symptoms explained by CC became non-significant after controlling for the influence of other predictors (Δ(*F*1,512) = 3.029, *p* = 0.08, Δ*R*^2^ = 0.003).

## Discussion

This study aimed to investigate whether the addition of certain cognitive processes to BA processes could predict a larger proportion of depressive symptoms and brooding. First, we explored the amount of variance in depressive symptoms that was explained by the target processes in BA treatment according to behavioral models (activation, behavioral avoidance, anticipatory pleasure, and brooding), by the target processes in CCT according to cognitive models (CC, AB_s_, and brooding), and by all processes together. Then we measured the relationships among these processes with path analysis in order to gain a comprehensive view of the interplay between them especially between activation and anticipatory pleasure and between CC, AB_s_ and brooding. Finally, we tested the amount of variance in depressive symptoms that was explained by the target processes in BA and by the target processes in CCT after controlling for the effect of the other predictors.

First, the analyses of the behavioral models revealed that activation, behavioral avoidance, anticipatory pleasure, and brooding are significant predictors of depressive mood. This result suggests that each process is a relevant therapeutic target for BA interventions. All the behavioral processes together explained a substantial amount of the variance in depressive symptoms (43%). In contrast, the analyses of the cognitive model revealed that only CC and brooding are significant predictors of depressive mood. Moreover, CC is no longer a significant predictor of depressive mood when the influence of other predictors is controlled for, as the hierarchical regression analysis, showed. Furthermore, our results did not support any claim that CC predicts ABs or brooding, or that ABs predict brooding. All the cognitive processes together explained 27% of the variance in depressive symptoms, with 25.6% explained by brooding and less than 1 and 2% explained by the ABs and CC, respectively. Analysis of the comprehensive model revealed that the combination of behavioral and cognitive models fit the data well but did not explain more of the variance in depressive symptoms or brooding than the behavioral models. These findings may corroborate the empirical data reported by Moshier and Otto (2017), which showed that CCT did not enhance the effect of a BA intervention on brooding and depression in a clinical sample. Of course, this should be weighed regarding cognitive functioning associated with clinical characteristics, including comorbidities of populations and must continue to be investigated.

Overall, given that none of the selected cognitive processes significantly predicts brooding, our findings do not corroborate the idea that brooding is partially due to deficits in working memory or AB_s_, as suggested by recent reports (Watkins and Roberts, 2020). Although unexpected, the lack of relationship between the monitoring of representations within working memory and brooding is consistent with a recent meta-analysis that reported null findings regarding the association between these processes (except for discarding cognitive function) in a sample of participants with and without depression diagnosis (Zetsche et al., 2018). Furthermore, the lack of relations between AB_s_ and brooding and AB_s_ and depressive symptoms is consistent with a recent cross-sectional research conducted in a clinically depressed, subclinically depressed and never-depressed sample (Krings et al., 2020) and even prospective research on AB_s_ and depressive symptoms in a sample of participants who were remitted from Major Depressive Disorder (Elgersma et al., 2019).

These null findings might be due to the inadequate reliability of the paradigm used. Indeed, the exogenous cueing task is associated with less than ideal level of psychometric properties, as suggested by the high standard error for the AB variances and the low split-half reliability of the AB indices. The use of eye tracking during the task to continuously monitor the focus of visual attention would be a more appropriate alternative to measure AB_s_. Furthermore, CC was measured by an updating task. However, different CC functions such as inhibition, shifting, or even discarding formerly relevant information from working memory could also be used to assess CC abilities (Zetsche and Joormann, 2011; Zetsche et al., 2018). The lack of relationships between AB_s_ and CC and depressive symptoms might also be explained by the heterogeneity of depression. There are numerous depressive symptoms and they represent distinct entities (e.g., some are good predictors of psychosocial impairment and others are not, or some are well predicted by stress and others are not; Fried and Nesse, 2014; Fried et al., 2015). Furthermore, AB_s_ and CC might be related to specific depressive symptoms or part of a network of related symptoms (Kraft et al., 2019). In addition, AB_s_ and CC might be related to other disturbed psychological processes not included in this model (e.g., interpretive bias, memory bias). Future research may benefit from exploring the interplay between these processes and specific depressive symptoms, as well as other disturbed psychological processes.

The behavioral path analysis models support the relevance of the behavioral model, showing that activation partially predicts anticipatory pleasure, which in turn predicts depressive symptoms. This result is in line with previous studies reporting that activation can influence reward anticipation and reward motivation in a subclinical depressed sample (Bakker et al., 2017) but also in an unselected sample of undergraduate students (Beevers and Meyer, 2002). In behavioral activation treatment, people are encouraged to become increasingly involved in goal-directed activities, which should increase the number of positive situations or events they experience, in order to improve their depression. Our findings suggest that this BA strategy may actually affects a significant proportion of depressive symptoms through its influence on anticipatory pleasure. This strategy acts partly as “reward exposure” and the repeated activation of reward networks normalizes the reward system. In addition to results reported by Bakker et al. (2017) and Beevers and Meyer (2002), other empirical data support this rationale, with findings suggesting that “Engage” therapy using exposure to meaningful activities helps to reduce depression in clinical samples (Alexopoulos et al., 2016, 2017). However, even if it is significant, it is important to note that activation explained only 6% of the variance in anticipatory pleasure. This result suggests that, to increase the efficacy of BA interventions, the treatment may include other therapeutic strategies that directly target anticipatory pleasure, in addition to activation. Recent empirical data suggest that enhancing the specificity and detail of episodic future thinking by increasing vividness and mental imagery represents a promising strategy to increase anticipatory pleasure in a clinically depressed sample (Hallford et al., 2020). Moreover, a recent study in healthy volunteers revealed that multisensory imagery of planned rewarding activities increased both anticipatory pleasure and engagement in these activities (Renner et al., 2019).

Some promising new treatments have recently emerged to enhance reward responsiveness in relation to anhedonia and depression. First, Positive Affect Treatment consists of an augmentation of a behavioral activation training module, a cognitive training module, and a compassion training module (Craske et al., 2016). This intervention is associated with an increase in positive affect, and depression for subjects suffering from anhedonia that lasted 6 months (Craske et al., 2019). Another treatment, Behavioral Activation Treatment of Anhedonia (BATA), includes several additional specific modules to BA (see Forbes, 2020; Nagy et al., 2020). BATA is associated with an improvement in reward processing for subjects suffering from anhedonia (Cernasov et al., 2021).

Finally, hierarchical regression analyses based on the magnitude of each predictor suggest that brooding was the best predictor of the variance in depressive symptoms, followed by behavioral avoidance, activation and then anticipatory pleasure. These findings support the relevance of BA processes as primary therapeutic targets. Because brooding is a good predictor of depressive symptoms, it is important to investigate empirically validated treatments that might target cognitive aspects of brooding and could serve as adjuncts to psychotherapy. One promising intervention targeting cognitive aspects related to brooding is memory specificity training, which targets autobiographical memory specificity (Martens et al., 2019). Concreteness training designed to teach individuals to become more concrete and specific in their thinking is another promising intervention that can be added to BA (Watkins and Moberly, 2009; Spinhoven et al., 2018). The unique contribution of selected processes is weaker than expected. Past studies reported a higher contribution of brooding with 46% of the variance in depressive symptoms explained by brooding in an unselected adults sample (Krings et al., 2020) and 17% in a subclinical sample of bereaved adults (Eisma et al., 2020). Behavioral avoidance explained 41% of the variance in depressive symptoms and activation explained 22% in an unselected adults sample (Krings et al., 2020). Past studies reported inconsistent results for cognitive control (Zetsche and Joormann, 2011) and to our knowledge no study have reported results on AB_s_ or anticipatory pleasure contributions. However, most of these past studies did not control for the common variance between these processes that may be significantly correlated. The minimal effects reported in our study may be explained by the control of this common variance. Future studies should estimate the unique contribution of depressive symptoms predictors as little is known yet in the literature.

### Limitations

Some limitations should be taken into account when interpreting these results. First, our findings use a cross-sectional design, making it impossible to examine causal relationships between variables or to be confident about the directionality of effects. The use of a non-clinical sample also limited the generalizability of our conclusions. Additionally, the data are associated with floor or ceiling effect of variables, including those of CC. Most participants did fairly well on the PASAT, which is not characteristic of psychiatric populations. As such, it would be difficult to detect relationships between the PASAT and outcome measures. However, the total sample of clinical participants was too small to reliably compare clinical and non-clinical depressed participants. Future studies should then explore the impact of these predictors on depressive symptoms in a clinical sample. Furthermore, path coefficients were examined in only one direction for the purpose of this study, but most factors may have reciprocal relationships and be mutually reinforcing (Roberts et al., 2017). In addition, the lack of results related to cognitive targets in our sample may mirror the heterogeneous symptoms characterizing depressive symptomatology but the total sample of participants was too small to reliably examine the interplay between the aforementioned processes and specific symptoms (e.g., fatigue, feeling guilty). Finally, the selected processes were theory-driven but were not exhaustive. The role of other relevant vulnerability processes in predicting depressive symptoms (e.g., consummatory pleasure, interpretive bias, memory bias, reappraisal) should be investigated in future studies.

## Conclusion

Our results suggest that behavioral models including activation, behavioral avoidance, brooding and anticipatory pleasure can explain much of the variance in depressive symptoms. Moreover, activation partially predicts anticipatory pleasure, which in turn predicts depressive symptoms. Our results also revealed that the cognitive model was relevant but CC and AB_s_ did not predict brooding or depressive symptoms. Consequently, they raise questions about the claim that AB_s_ and CC figure prominently in the maintenance of depressive symptoms and brooding. A comprehensive model including every process did not explain more of the variance in brooding or depressive symptoms than the behavioral models suggesting that cognitive training may not be a promising add-on treatment to behavioral activation in our participants with such clinical characteristics. Our findings cast some doubt on the robustness of earlier findings in the field, and future research should carefully investigate the cognitive models of depression. Findings also indicate further investigation of mechanisms of change in BA and CCT treatments in depressed patients is warranted/indicated at this time.

## Author’s Note

AC is a member of the Federative Research Structure in Health, Prevention, Quality of Life of the Université Savoie Mont Blanc.

## Data Availability Statement

The datasets presented in this study can be found in online repositories. The names of the repository/repositories and accession number(s) can be found at: https://osf.io/hfj8a/.

## Ethics Statement

The studies involving human participants were reviewed and approved by Central ethics committee at University of Liège. The patients/participants provided their written informed consent to participate in this study.

## Author Contributions

AK and AC: data collection and article preparation. JS and SB: article preparation. All authors contributed to the article and approved the submitted version.

## Conflict of Interest

The authors declare that the research was conducted in the absence of any commercial or financial relationships that could be construed as a potential conflict of interest.

## Publisher’s Note

All claims expressed in this article are solely those of the authors and do not necessarily represent those of their affiliated organizations, or those of the publisher, the editors and the reviewers. Any product that may be evaluated in this article, or claim that may be made by its manufacturer, is not guaranteed or endorsed by the publisher.

## Footnotes

^1^To have more information about this scale, please contact the first author of the manuscript.

^2^The central ethics committee at University of Liège located at CHU Sart-Tilman, B35, 4,000 Liège approved this study in 2018 (Belgian number: B707201629390, reference number: 2016–215).

^3^All analyses were also conducted with diagonally weighted least squares method, as the choice of parametric or non-parametric statistical method may be controversial. Analyses revealed similar results and conclusions.
